# Supplementary material for: Diet and Blood Concentrations of Essential and Non-Essential Elements among Rural Residents in Arctic Russia
Source: Nutrients. 2022 Nov 24;14(23):5005. doi: 10.3390/nu14235005 (PMC9738226; doi:10.3390/nu14235005)
Supplement: Supplementary file 1 [file nutrients-14-05005-s001.zip › nutrients-2019251-supplementary.pdf]

Supplement Table S1 The arithmetic and geometric means and minimum and maximum concentrations of elements in whole blood (µg/L) according to reported (yes/no) consumption of different traditional food items, being a hunter, catching fish or smoking among the participants.

**Anadromous fish**	**Yes N=238**	**No N=58**	**p**
**As**[a]	5.3 (0.5-163)	2.9 (0.5-40.0)	0.005
**Semi-anadromeous fish**	N=154	N=142	
**Mn**	13.1 (5.4-28.5)	14.4 (4.3-35.0)	0.04
**Hg**	5.6 (0.5-24.3)	4.7 (0.3-23.8)	0.07
**Pb**[a]	22.1 (5.4-356)	26.7 (5.4-281)	0.047
**Marine fish**	N=198	N=98	
**Mn**	14.2 (5.3-34.5)	12.9 (4.3-35.0)	0.04
**As**[a]	6.8 (0.5-163)	2.3 (0.5-62.4)	<0.001
**Freshwater fish**	N=155	N=141	
**Mn**	12.8 (4.3-28.5)	14.8 (5.1-35.0)	0.001
**Cu**	1.12 (0.60-1.75)	1.08 (0.58-2.40)	0.09
**Zn**	8.6 (4.1-13.7)	9.1 (4.9-14.5)	0.02
**Hg**	6.2 (0.5-24.3)	4.0 (0.3-21.4)	<0.001
**Pb**[a]	21.5 (6.2-356)	27.7 (5.2-281)	0.008
**As**[a]	3.7 (0.5-163)	6.3 (0.5-140)	<0.001
**Reindeer**	N=240	N=56	
**Mn**	13.5 (4.3-35.0)	14.8 (5.3-28.6)	0.10
**Co**[a]	0.54 (0.25-2.49)	0.50 (0.30-0.91)	0.08
**Cu**	1.11 (0.58-2.40)	1.05 (0.73-1.97)	0.10
**Other local meat**	N=41	N=255	
**Hg**	6.2 (1.4-18.4)	5.0 (0.3-24.3)	0.08
**Se**	131 (93.7-192)	124 (84.7-227)	0.03
**Goose**	N=242	N=53	
**Zn**	8.7 (4.1-13.7)	9.3 (5.7-14.5)	0.050
**Pb**[a]	25.5 (5.2-356)	19.2 (5.4-164)	0.02
**As**[a]	5.1 (0.5-163)	3.4 (0.5-40.1)	0.06
**Other local birds**	N=141	N=155	
**Co**[a]	0.51 (0.25-1.73)	0.54 (0.29-2.49)	0.07
**As**[a]	5.8 (0.5-163)	3.9 (0.5-140)	0.01
**Mushroom**	N=86	N=210	
**As**[a]	6.1 (0.5-143)	4.2 (0.5-163)	0.03
**Se**	129 (84.7-227)	123 (88.1-192)	0.04
**Blueberry**	N=143	N=152	
**Mn**	13.0 (4.3-35.0)	14.4 (4.5-33.5)	0.03
**Zn**	8.6 (4.1-14.5)	9.1 (4.8-13.1)	0.01
**Hg**	5.6 (0.3-24.3)	4.7 (0.5-23.8)	0.06
**Pb**[a]	22.2 (5.6-356)	26.4 (5.2-281)	0.06
**As**[a]	4.0 (0.5-163)	5.5 (0.5-140)	0.04

**Cloudberry**	N=238	N=57	
**Mn**	14.1 (4.3-35.0)	12.3 (5.1-24.0)	0.006
**Cowberry**	N=205	N=90	
**Co**[a]	0.54 (0.26-2.5)	0.50 (0.25-0.92)	0.07
**Pb**[a]	21.7 (5.4-356)	31.3 (5.2-281)	0.001
**Other berries**	N=82	N=213	
**As**[a]	5.9 (0.5-16.3)	4.3 (0.5-140)	0.08
**Being a hunter (Missing data!)**	N=55	N=202	
**Co**[a]	0.49 (0.25-1.7)	0.54 (0.27-2.5)	0.02
**Cu**	1.04 (0.58-1.43)	1.11 (0.69-1.97)	0.04
**Pb**[a]	39.8 (8.0-356)	20.8 (5.2-218)	<0.001
**Catching fish**	N=130	N=148	
**Co**[a]	0.50 (0.25-1.2)	0.56 (0.28-2.5)	0.001
**Cu**	1.07 (0.58-2.40)	1.13 (0.69-1.97)	0.04
**Pb**[a]	28.8 (5.4-281)	21.0 (5.6-356)	0.001
**Smoking**	N=97	N=189	
**Cd**[a]	0.76 (0.13-4.2)	0.22 (<DL-3.0)	<0.001
**Pb**[a]	31.3 (6.8-218)	21.4 (5.2-356)	<0.001

[a]geometric mean
